# A theoretical approach to spot active regions in antimicrobial proteins

**DOI:** 10.1186/1471-2105-10-373

**Published:** 2009-11-11

**Authors:** Marc Torrent, Victòria M Nogués, Ester Boix

**Affiliations:** 1 Dpt. Bioquímica i Biologia Molecular, Fac. Biociències, Universitat Autònoma de Barcelona, 08193 Cerdanyola del Vallès, Spain

## Abstract

**Background:**

Much effort goes into identifying new antimicrobial compounds able to evade the increasing resistance of microorganisms to antibiotics. One strategy relies on antimicrobial peptides, either derived from fragments released by proteolytic cleavage of proteins or designed from known antimicrobial protein regions.

**Results:**

To identify these antimicrobial determinants, we developed a theoretical approach that predicts antimicrobial proteins from their amino acid sequence in addition to determining their antimicrobial regions. A bactericidal propensity index has been calculated for each amino acid, using the experimental data reported from a high-throughput screening assay as reference. Scanning profiles were performed for protein sequences and potentially active stretches were identified by the best selected threshold parameters. The method was corroborated against positive and negative datasets. This successful approach means that we can spot active sequences previously reported in the literature from experimental data for most of the antimicrobial proteins examined.

**Conclusion:**

The method presented can correctly identify antimicrobial proteins with an accuracy of 85% and a sensitivity of 90%. The method can also predict their key active regions, making this a tool for the design of new antimicrobial drugs.

## Background

Host defence anti-microbial proteins and peptides are important participants of the innate immune response in most multicellular organisms [1]. The innate immune system comprises the cells and mechanisms that defend the host from infection by other organisms in a non-specific manner. Unlike the adaptive immune system, the innate immune system does not confer a long-lasting or protective immunity to the host, but is thought to constitute an evolutionarily older defence strategy. It remains the dominant immune system in plants, fungi and insects, and plays a crucial role during the first steps of infection in multicellular organisms.

One of the major achievements of medicine was the development of antibiotics, which can kill a broad spectrum of microorganisms. Unfortunately, the emergence of antibiotic resistance has become a clinical threat [2,3]. Cationic proteins and peptides that are involved in innate immunity represent an alternative strategy to conventional antibiotics [4]. A considerable variety of peptides with different size and structure are associated with antimicrobial activity in eukaryotic hosts. Antimicrobial cationic peptides have some common structural characteristics. They are composed of 12-50 amino acids, with 2-9 cationic residues and up to a 50% hydrophobic amino acids [5]. Many peptides do not adopt a well-defined secondary structure in aqueous solution, but adopt a specific secondary structure upon binding to acidic phospholipids or within lipid bilayers [6].

A major limitation in the design of new antimicrobial peptides lies in the difficulty in finding new structures with low toxicity for the host and a broad spectrum of action against pathogens. An important group of antimicrobial peptides are derived from fragments released by proteolytic cleavage of larger proteins or derived from known antimicrobial regions of proteins involved in the innate immune system, such as the C-terminal domain of cathelicidins [7] or the N-terminus of bactericidal/permeability increasing protein (BPI) [8,9]. Additionally, antimicrobial peptides often display immunomodulation properties which can be applied in the design of new drugs for the treatment of immune system disfunctions, such as autoimmune diseases [10]. Knowledge of the key regions in antimicrobial proteins is of great importance in deriving antimicrobial peptides.

Following this approach, we present a method of predicting potentially active regions of bactericidal proteins that can serve as templates to develop agents against antimicrobial resistance.

## Results and discussion

### Method description

Experimental data based on high throughput screening and database mining techniques show that certain residues are preferred in antimicrobial peptides. Hilpert et al. screened all amino acid substitutions for each position of a 12-mer peptide and tested all substitutions for activity, which was measured as the bactericidal half-maximal inhibitory concentration (IC_50_) [11].

Bactenecin (also called bovine dodecapeptide), the antimicrobial peptide used by Hilpert et al., was discovered in neutrophils and is active against both Gram-negative and Gram-positive bacteria [12]. These authors used a variant of bactenecin called Bac2A (RLARIVVIRVAR-NH_2_), where internal disulfide bridges had been eliminated. Bac2A had a high antimicrobial activity against both Gram-positive and Gram-negative bacteria, and was used as a template to test the effect of each amino acid substitution on the peptide's activity. The IC_50 _value against the *Pseudomonas aeruginosa *was recorded for each synthetic peptide. Based on the screening results, we calculated a propensity value for each amino acid.

Although the bacterial strain for the high-throughput assay is Gram-negative, the peptide has also been tested against other strains [12]. The results have demonstrated that Bac2A is a peptide with broad antimicrobial action, which is also active against Gram-positive strains and fungi. No differences were found between proteins with distinct antimicrobial properties in both the training and testing datasets. Our selected dataset covers a wide range of antimicrobial proteins that have activity on distinct strains from Gram-negative and Gram-positive bacteria, and the prediction data discard any potential bias. Nevertheless, some discrepancies may arise between the predicted and experimental regions if a specific determinant is found in a protein conferring some specificity for a particular microorganism or strain.

The calculated propensity index (Table 1), deduced from the corresponding IC_50 _value for each amino acid substitution, makes a good reference for the assessment of the protein sequence determinants. Since low IC_50 _values correspond to high antimicrobial activity, amino acids with a lower PV value represent the most favoured to take the part of an antimicrobial peptide. Positively charged residues (R, K) and some hydrophobic residues (W, Y, I, V) were favoured and present a low propensity index, whereas negatively charged residues are unfavoured and show a high propensity index. Positively charged residues would be necessary to direct antimicrobial proteins to the negatively charged bacterial cell wall and cytoplasmic membrane of bacteria, where they exert their antimicrobial effect [13]. Hydrophobic residues would be needed to interact with lipophilic regions of lipid bilayers in order to create pores or other destabilizing structures that lead to membrane depolarization or local disruption and eventually bacterial cell death [14]. Interestingly, W has the lowest PV value among the hydrophobic residues, whereas L has the highest value, while I and V rather than L, are preferred. In fact, W residues are known to be important for antimicrobial peptide action [15].

**Table 1:** Bactericidal propensity values (PV) for each amino acid.

Amino acid	PV value
R	0.106
K	0.111
C	0.165
W	0.172
Y	0.185
I	0.198
V	0.200
H	0.202
N	0.240
T	0.242
F	0.246
L	0.246
Q	0.248
G	0.265
M	0.265
S	0.281
A	0.307
P	0.327
E	0.449
D	0.479

Calculations are based on the average half maximal inhibitory concentration, as detailed in the *Methods *section.

Because bactericidal action is generally located in a specific region, a sliding window system of 7 residues was chosen for the screening approach. To improve the predictive accuracy, 3 predictive lengths of 10, 12 and 14 residues were evaluated. For each length, the optimal number of permissible gaps was tested (2, 3 or 4 gaps). For each combination of parameters, an ROC curve was constructed, and the accuracy, sensitivity and selectivity of the method calculated in order to select the best parameters [see Additional file 1: Supplemental Figures S1, S2 and S3]. Optimal results were obtained using a predictive length of 12 amino acids allowing 2 gaps (Table 2). For these conditions, the AvPV threshold value was 0.225; thus residues with an AvPV <0.225 were considered favourable whereas residues with an AvPV >0.225 were considered unfavourable. Allowing a higher gap number drastically lowered both the specificity and sensitivity, so only 2 gaps were allowed. Using the selected cut-off value (a predictive length of 12 residues with 2 allowed gaps) we achieved an accuracy value of 85%, a sensitivity of 90%, and a specificity of 80% (Table 2).

**Table 2:** Evaluation of the tested parameter values for the selection of optimal predicting length and gap inclusion.

Predictive length (Gaps)	Sensitivity	Specificity
10 (2)	92%	78%
10 (3)	86%	82%
10 (4)	92%	78%
**12 (2)**	**90%**	**80%**
12 (3)	88%	82%
12 (4)	86%	84%
14(2)	86%	84%
14 (3)	86%	84%
14 (4)	84%	86%

Although this method detects antimicrobial regions with high accuracy, it may not be adequate in predicting antimicrobial regions with a high content of some specific amino acids, e.g. proline. Likewise, F residues are considered to favor antimicrobial activity [16], but this residue has a relatively high calculated index. Our method has been constructed with data provided from a peptide with particular amino acid content, and this may slightly alter the effect of some of the substitutions and the final output of their assigned propensity values. This may increase the number of target proteins missed, but do not alter the significance of the positive hits. In any case, the propensity indexes can be recalculated as other experimental data become available, to update and improve the method prediction power.

### Method testing and implementation

The proposed method was applied to a set of 100 proteins (50 bactericidal proteins and 50 non-bactericidal). Representative members of the main antimicrobial protein families described in the literature were included, and the results were compared and discussed with the available experimental data (Table 3).

**Table 3:** Detail of the predicted sequences from the studied antimicrobial proteins.

Proteins	Identified sequences	Experimental data reported
hCAP 18	RKSKEKIGKEFKRIVQRIKD	LLGDFF**RKSKEKIGKEFKRIVQRIKD**FLRNLVPRTES [17]
CAP 11	LGGRRFRRMVGLRKKFRKTRKRIQKLGRKIGKTGRKVWKAW	G**LRKKFRKTRKRIQKLGRKIGKTGRKVWKAW**REYGQIPYPCRI [19]
CRAMP	RFKKKISLRAGLLRKGG.... EKLKKIGQKIKNFFQ SVRFRVKETV	**ISLRAGLLRKGG**EKIG**EKLKKIGQKIKNFFQ**KLVPQPE [21]
BPI	SVHVHISKSKVGWLIQLFHK NCIKISGKWKAQKRFLK	**KSKVGWLIQLFHK**K [23] ISNA**NIKISGKWKAQKRFLK**MSGNFDLSI*
Histone H2A	GRGKQGGKVRAKAKTRS//GRVHRLLRKG KKTRIIPRHLQL	**SGRGKQGGKVRAKAKSRSSR**AGLQFPV**GRVHRLLRKG**NY [25]
Lysozyme	AKSRWYNQTPNRAKRVITTFRT	WDEAAVN**LAKSRWYN**[27]
Azurocidin	SGGRLSRFPRFVNV PNNVCTGVLTRRGGI	50% sequence identity with protegrin antimicrobial peptides
α-defensin	YGTCIYQGRLWAFCC	R**YGTCIYQGRLWAFS**[36]
β-defensin	TLQKYYCRVRGGRCAVLSC KCSTRGRKCCRRKK	GIIN**TLQKYYSRVRGGR** **KSSTRGRKSSRRKK**[37]
θ-defensin	GLRCICTRGFCRLL	**RCICTRGFCRLL**[39]
Magainin precursor	IGKFLHSAKKFGK	G**IGKFLHSAKKFGK**AFVGEIMNS [40]
Moronecidin	HIFRGIVHVGKTIHRLVT	FFH**HIFRGIVHVGKTIHKLVT**G [44]
Pleurocidin	FFKKAAHVGKHVG	GWGS**FFKKAAHVGKHVG**KAALTHYL [45]
Bacteriocin enterocin	SCNKKGSCPGVKYGKKLGG	KYYGNGV**SCNKKGCSV**[49]
Helveticin J	VVQKGNVGSKYVYGLQLRKGA	No active peptide reported
Sarcotoxin IA	WLKKIGKKIERVGQ	W35 is described as a key residue in endotoxin neutralizing activity [52]
RNase 3	INNYRWRCKNQNTFLR VNVCGNQSIRCPHNRTLNNCHRSRFRVPL	TIAMRA**INNYRWRSKNQNTFLR**[61]
RNase 7	NINKHTKRCK SHGRVSLTMCKLTSGKYPNCRYKEKRQNKSYVVA	No active peptide reported
SPAG 11K	QLLRHPVKRAPIIRRIP	N -terminus suggested [68]
Hepcidin	LCRFCCKCCRNKGCGYCCKF	No active peptide reported
Ace AMP1	ICPRVNRIVT VNTRNLRRAACRCLVGVVNRNPGLRRNPR RNTFVRPFWWRPRIQCGRI	No active peptide reported

* United States Patent n° 5830860

The first column includes the identified sequences by the prediction approach. The corresponding active peptides previously described in the literature and tested experimentally are included in the adjacent column for comparison. Matching residues between the identified and experimentally active sequences are underlined and highlighted in bold.

Most of the known antimicrobial proteins were correctly identified and their active region was accurately predicted, when adequate information was available (Table 3).

To probe its reliability, the method was also applied to a positive testing dataset containing 20 antimicrobial proteins. It predicted 90% of the proteins in it. A negative testing dataset was also analyzed and 81% of the proteins were correctly identified as non-antimicrobial proteins. The results obtained are in good agreement with those presented for the training dataset [see Additional file 1: Supplemental Tables S1 and S2].

We describe below the main representative families of antimicrobial proteins that were examined, together with an exhaustive comparison between the predicted identified sequences and the experimentally active reported regions (Table 3).

*Cathelicidins *comprise a family of mammalian proteins expressed in epithelial and myeloid cells, involved in the innate immune response [10]. Cathelicidins contain a C-terminal cationic antimicrobial domain that becomes active on release from the N-terminal region of the holoprotein [17]. The most studied form is the human cathelicidin antimicrobial protein of 18 kDa (hCAP18). Its C-terminus (LL-37) has a wide spectrum of antimicrobial activity and other biological activities [18]. Analysis of the human cathelicidin sequence leads to the prediction that the LL-37 peptide region is responsible for antimicrobial activity (*hCAP 18*, Table 3).

This family comprises other known antimicrobial peptides active against a great variety of bacteria, including CAP11 or CRAMP, with a very low sequence identity and distinct assigned active regions. In all cases, antimicrobial regions predicted span the sequence corresponding to experimentally reported active peptides (*CAP11 and CRAMP*, Table 3) [19,20].

*Bactericidal/permeability-increasing protein (BPI) *is a 456-residue cationic protein stored in the polymorphonuclear leukocytes primary granules [21]. During phagocytosis and degranulation, proteases cleave BPI in the 236-241 region, releasing the fragment corresponding to the N-terminus end [8]. This fragment is responsible for its antibacterial activity [22]. Our method predicts 2 potential antimicrobial regions in the N-terminus of, in good agreement with the reported experimental data (*BPI*, Table 3). One of these regions (rBPI21) is reported to be clinical useful [23]. The other region is a potent endotoxin neutralizing peptide, thus providing a potential therapeutic value for peptides tested (USA Patent 5830860).

*Histone H2A *is one of the 5 main histone proteins involved in the structure of chromatin. Buforin I is a 39 amino acids peptide encoded by the same gene as histone H2A. A specific protease responsible for the generation of buforin I from histone H2A is in the crude extracts of the toad stomach, suggesting the presence of a specific functional regulation mechanism which converts toad histone H2A to buforin I. Moreover, a more potent antimicrobial peptide of 21 amino acids, buforin II, derived from buforin I [24,25] shows high antimicrobial activity against a broad spectrum of microorganisms. The histone H2A sequence screening analysis shows 2 potential antimicrobial regions. The first predicted sequence is part of buforin II sequence (*Histone H2A*, Table 3). There seems to be no report of any experimental evidence for the second region predicted. However, the ratio between charged and hydrophobic residues makes this peptide a good target to test.

*Lysozyme *is an antibacterial protein with activity against Gram-positive and Gram-negative bacteria; muramidase activity is considered responsible for its bactericidal activity [26]. However, the denatured protein is also active, showing that lysozyme retains its antimicrobial activity when muraminidase activity is absent. In addition, some derived peptides from the lysozyme C-terminus, e.g. peptide A4 (residues 143-155), also retain antimicrobial activity [27,28]. These results agree with our prediction about this region in bacteriophage T4 lysozyme (*Lysozyme*, Table 3), further supporting the hypothesis that bactericidal activity of lysozyme is not uniquely attributed to its muramidase activity.

*Serprocidins *are 25-37 kDa serine proteases localized in neutrophil granules with cytotoxic activity against both Gram-negative and Gram-positive bacteria [29].

There is no evidence for a defined antimicrobial region in azurocidin, the human serprocidin. Although a peptide comprising the region 20-44 has been suggested [30], subsequent studies found no direct correlation between this segment and antimicrobial activity [31].

Our results also suggest that 2 other regions are involved in the bactericidal activity of azurocidin. Of special interest is the predicted antimicrobial region SGGRLSRFPRFVNV that shows a 53% of identity with protegrins (*Azurocidin*, Table 3), a group of antimicrobial peptides in porcine leukocytes that exhibit *in vitro *broad-spectrum antimicrobial activity [32,33].

*Defensins *are a family of antimicrobial peptides [34] showing antimicrobial activity against Gram-negative and Gram-positive bacterial strains, fungi, and some parasites and enveloped viruses. Defensins can be classified by origin and structure. Vertebrate α and β defensins share a common fold and are mainly distinguished according to their disulfide bridge pattern, while θ defensins are α-derived cyclic peptides.

Our results predict a main antimicrobial region for α-defensins in agreement with published data (*α-defensin*, Table 3). The prediction screening points to the C-terminus of α-defensins as the key region that retain antimicrobial activity. Recent studies on the antimicrobial activity of α-defensins also support this hypothesis [35].

In analysing human β-defensin, we identified a potential active sequence at both the N- and C-termini. The C-terminal sequence of β-defensins retains the antimicrobial capacity of the whole protein against Gram-negative bacteria. Moreover, the N-terminal sequence seems to be necessary for activity against Gram-positive bacteria and fungi. Thus, the contribution of both N- and C-termini is not completely understood, but seems necessary to have broad spectrum antimicrobial activity [36,37]. These findings coincide with the prediction results, suggesting that the whole protein is involved in its antimicrobial action (*β-defensin*, Table 3).

Finally, we have predicted the active domain described in *Rhesus macaque *θ-defensin (*θ-defensin*, Table 3) [38,39].

*Magainins *are a class of antimicrobial peptides discovered in the skin of *Xenopus laevis *[40-42]. In analyzing the magainin precursor that contains 5 copies of the active peptide, and our method can identify the active segments (*Magainin precursor*, Table 3).

*Piscidins *were the first antimicrobial cationic peptides to be isolated from the mast cells of striped bass fish [43]. We have analyzed the potential antimicrobial regions of 2 proteins: moronecidin and pleurocidin *(Moronecidin, pleurocidin*, Table 3). The predictions are in good agreement with the experimentally tested peptides, which, in both cases, correspond to the mature peptide released after the cleavage of the propeptide region from the protein [44,45].

*Bacteriocins*, antimicrobial proteins or peptides produced by bacteria, are expressed in lactic acid bacteria (LAB) [46,47].

We have analyzed bacteriocins, which have been the most studied peptides. For bacteriocin enterocin CRL35, the region predicted at its N-terminus overlaps with some sequences tested in literature (*Bacteriocin enterocin*, Table 3) [48]. We have also analyzed bacteriocin helveticin-J, and identified a potential antimicrobial region, but there is no data on the region responsible for its activity to corroborate this prediction (*Helveticin-J*, Table 3).

*Sarcotoxin IA *is a cecropin-like polycationic peptide that is active against a wide range of both Gram-positive and Gram-negative bacteria [49]. The natural 62 amino acid precursor of sarcotoxin is processed, resulting in a 39 amino acid long mature peptide [50]. No region has been described for sarcotoxin IA as primarily responsible for its antimicrobial action. However, key residues have been identified as important in the bactericidal activity of sarcotoxin IA, specifically W35, implicated in endotoxin- neutralizing activity [51]. W35 residue lies in the predicted potential antimicrobial region (*Sarcotoxin IA*, Table 3). Moreover, our predicted sequence shows 45% amino acid identity with salmocidins, an antimicrobial peptide group isolated from *Salmo gairdneri*, which has still to be characterized (deposited in SwissProt P81369).

*Antimicrobial ribonucleases *The RNase A family includes RNases with cytotoxic and antipathogen properties [52-54].

The eosinophil cationic protein (ribonuclease 3 or ECP) is a human host defence ribonuclease involved in inflammatory processes mediated by eosinophils [55,56]. ECP is a potent cytotoxic molecule, with bactericidal and helminthotoxic properties [57]. ECP antimicrobial activity is dependent on its action at the cytoplasmic membrane and bacteria wall [58,60]. Our recent experimental data confirm that the first predicted sequence (*RNase 3*, Table 3) is involved in the protein bactericidal activity [61]. Screening of the membrane lysis and bactericidal activity of RNase 3 derived peptides corroborated that the protein N-terminus region retains most of its antimicrobial activity. The role of the second predicted region remains to be determined.

Ribonuclease 7 is an antimicrobial protein expressed in skin, liver, kidney, skeletal muscle and heart. RNase 7 has a high antimicrobial activity against *P. aeruginosa *and *P. Pastoris*, and a lower effect in *S. aureus *and *E. coli *cells [62-64]. RNase 7 site-directed mutagenesis studies indicate that some lysine clusters are necessary for the protein antimicrobial action, although not every cluster is of equal importance [64]. In this context, the regions predicted by our theoretical approach include a great number of lysine residues, giving them a high cationic content (*RNase 7*, Table 3). Nevertheless, the selected sequences present a low hydrophobic residue content in contrast to most known antimicrobial regions. As the RNase 7 antibacterial mechanism has not been identified, we cannot yet explain these results.

## Conclusion

Recently, much attention has been paid to develop computational methods to screen and synthesize antimicrobial peptides. High throughput screening tests provide a powerful tool to design predictive methods. A method using high throughput screening that predicts antimicrobial action of peptides in *P. aeruginosa *has been published [65]. Other screening and bioinformatics approaches in the quest for new antimicrobial agents have recently been described. Lata et al. [66] designed a program to predict antimicrobial peptides, based in the observation that certain types of residues are preferred over others, particularly at the N- and C- termini. Using a support vector machine, this approach is a powerful tool with which to predict or identify antimicrobial peptides.

Our method differs from previously reported predictive algorithms because it applies experimentally derived high throughput screening values obtained in synthetic peptides to analyse protein sequences. Few attempts have been made to analyse bactericidal proteins to identify the structural determinants for their mechanism of action.

The proposed predictive screening approach has been applied to the main characterized antimicrobial protein families, allowing a direct comparison between the identified sequences and previous experimental data. We corroborate that the predicted sequences mostly match the regions experimentally reported by others. Moreover, our own experimental data testing a RNase 3 peptide collection was also proven successful [61]. Other antimicrobial proteins (SPAG 11K, Hepcidin and Ace AMP1) have also been analysed and successfully predicted as antimicrobial proteins (Table 3). In these cases, no other experimental results have been reported, which precludes direct confirmation of the capacity of the prediction method. However, the results presented here provide an opportunity to study predicted peptides. To further optimize the method prediction power we are planning to update the provided indexes as new high throughput screening results become available.

Thus, this method can give a first approach in spotting the key regions of bactericidal proteins that give them their activity. The selected regions may provide a useful starting point in the development of new antimicrobial peptide derived drugs.

## Methods

### Sequence analysis

Protein sequences were obtained from the Swiss-Prot/TrEMBL Database http://www.ebi.ac.uk/swissprot/. Pair-wise and multiple alignments were made with *ClustalW *http://www.ebi.ac.uk/Tools/clustalw2. Multiple alignments with known antimicrobial peptides were performed using the Antimicrobial Peptide Database http://aps.unmc.edu/AP/main.php/[67].

### Data processing and model design

To predict potential antimicrobial regions, a bactericidal propensity index value (PV) was calculated for each amino acid. To ascribe a PV value to each, we took the experimental data of Hilpert et al. [11] where a complete library of 12 amino acid peptides was generated, starting from a template based on a linearized variant of bactenecin, a bovine antimicrobial peptide (Bac2A). From a high-throughput screening assay, activity against *Pseudomonas aeruginosa *for each amino acid substitution was tested and the bactericidal inhibitory concentration (IC_50_) value for each peptide was estimated [11]. Using these experimental data, the average minimal inhibitory concentration (called the propensity value, PV) was calculated for each residue (Table 1). Each propensity value corresponds to the arithmetical average between the 12 positions tested in the peptide Bac2A. The standard error associated with each score has an constant value of approximately 0.05.

The screening for antimicrobial regions is based on the calculated bactericidal propensity values per amino acid (PV), using the IC_50 _experimental values, as already discussed. The method calculates the average propensity value (AvPV) over a sliding window of the length of 7 residues and assigns this value to the central residue inside the window. The window size was selected to ensure the best signal/noise ratio for the tested sequences. The use of smaller window size may overestimate some regions, whereas using larger window sizes may lose some information.

A protein is classified as antimicrobial if it has at least one antimicrobial region. To define a region in a sliding window system, 2 parameters were considered: the minimal length of the region (called the predictive length) and the allowed gap inclusion. In this case, 3 different predicting lengths of 10, 12 and 14 residues were tested, making allowance for 2, 3 or 4 gaps in each case.

### Protein datasets

A training dataset of 50 antimicrobial proteins, comprising some of the best characterized antimicrobial protein families in the literature, was selected for evaluation. Some of the proteins sequence determinants have been reported experimentally, allowing a further checking of predicted results and experimental data. To complete the training dataset, 50 non-antimicrobial proteins recorded in the Swiss-Prot database were included. These proteins were randomly selected among those reported as soluble and having between 50 and 200 amino acid residues.

To assess the predictability of the method, a positive testing dataset has also been constructed, containing 20 antimicrobial proteins verified and annotated as antimicrobial according to Swiss-Prot. Another dataset, containing 20 soluble proteins has been constructed to provide a negative testing dataset. No proteins belonging to the training dataset were included in both cases [see Additional file 1: Supplemental Tables S1 and S2].

### Evaluation of the method

To assess the performance of the method and determine the best cut-off AvPV value for each case, we used the Receiver Operating Characteristic (ROC) curves, considering the parameters described below:

True positive (TP) and true negative (TN) are correctly predicted as antimicrobial proteins and non-antimicrobial proteins, respectively. False positive (FP) and false negative (FN) are wrongly predicted antimicrobial proteins and predicted non-antimicrobial proteins, respectively. MCC is the Matthew's correlation coefficient.

For each case, we constructed an ROC curve and determined its parameters as described in Table 2. Once the best ROC curve had been gained, the cut-off AvPV value was evaluated and the protein sequences were assayed using these values.

## Authors' contributions

MT designed the method, analyzed the data, and drafted the manuscript. EB participated in the data analysis, results discussion, and manuscript writing. VMN participated in the results discussion, critical reading and correction of the manuscript. All authors read and approved the final manuscript.

## Supplementary Material

Additional file 1 **Supplementary tables and figures**. The data provided includes the statistical ROC analysis and a detailed description of the testing datasets used to validate the method. Click here for file
