# Supplementary material for: A theoretical approach to spot active regions in antimicrobial proteins
Source: BMC Bioinformatics. 2009 Nov 11;10:373. doi: 10.1186/1471-2105-10-373 (PMC2780422; doi:10.1186/1471-2105-10-373)
Supplement: Additional file 1 — Supplementary tables and figures. The data provided includes the statistical ROC analysis and a detailed description of the testing datasets used to validate the method. [file 1471-2105-10-373-S1.doc]

**Supplementary Material**

**Manuscript Title: Theoretical approach to spot active regions in antimicrobial proteins.**

# Authors: Marc Torrent, Victòria M. Nogués and Ester Boix

Table S1. Negative testing dataset results.

| Protein ID | Predicted | Observed |
| --- | --- | --- |
| P56508 | N | N |
| Q05676 | N | N |
| P40204 | N | N |
| P41806 | N | N |
| P22289 | N | N |
| P37299 | N | N |
| P07215 | N | N |
| Q6Q547 | P | N |
| P40422 | N | N |
| P03094 | P | N |
| P69852 | N | N |
| Q12287 | N | N |
| Q3E764 | P | N |
| P04039 | N | N |
| Q6Q5K6 | P | N |
| P01094 | N | N |
| P01095 | N | N |
| P38636 | N | N |
| Q6Q546 | N | N |
| P81451 | N | N |

Specificity: 80 %

Negative (N) and positive (P) hits for each polypeptide are indicated, as predicted by the theoretical approach (Predicted) and reported from experimental evidence in the literature (Observed).

Table S2. Positive testing dataset results.

| Protein ID | Predicted | Observed |
| --- | --- | --- |
| P80032 | P | P |
| P81058 | P | P |
| P81592 | P | P |
| P01376 | P | P |
| P60030 | P | P |
| Q93X17 | P | P |
| Q95NH6 | P | P |
| Q07932 | P | P |
| P06833 | P | P |
| P37363 | N | P |
| Q7M249 | P | P |
| O76145 | P | P |
| P22749 | P | P |
| P32195 | P | P |
| Q6IV20 | P | P |
| B5LUR0 | P | P |
| Q25054 | N | P |
| Q29075 | P | P |
| P14213 | P | P |
| P82358 | P | P |

Sensitivity: 90 %

Negative (N) and positive (P) hits for each polypeptide are indicated, as predicted by the theoretical approach (Predicted) and reported from experimental evidence in the literature (Observed).

Figure S1. Statistical analysis of the results obtained using a 10 amino acid predictive length and different gap inclusion.

Figure S2. Statistical analysis of the results obtained using a 12 amino acid predictive length and different gap inclusion.

Figure S3. Statistical analysis of the results obtained using a 10 amino acid predictive length and different gap inclusion.
